# Nano-Formulations for Pulmonary Delivery: Past, Present, and Future Perspectives

**DOI:** 10.3390/pharmaceutics16020161

**Published:** 2024-01-24

**Authors:** Siyuan Peng, Wenhao Wang, Rui Zhang, Chuanbin Wu, Xin Pan, Zhengwei Huang

**Affiliations:** 1 School of Pharmaceutical Sciences, Sun Yat-sen University, Guangzhou 510275, China; pengsy23@mail2.sysu.edu.cn (S.P.); wangwh37@mail2.sysu.edu.cn (W.W.); zhangr358@mail.sysu.edu.cn (R.Z.); 2 College of Pharmacy, Jinan University, Guangzhou 510632, China; chuanbinwu@jnu.edu.cn

**Keywords:** inhalable nano-formulations, inhalation devices, industrialization, nebulizers

## Abstract

With the development of nanotechnology and confronting the problems of traditional pharmaceutical formulations in treating lung diseases, inhalable nano-formulations have attracted interest. Inhalable nano-formulations for treating lung diseases allow for precise pulmonary drug delivery, overcoming physiological barriers, improving aerosol lung deposition rates, and increasing drug bioavailability. They are expected to solve the difficulties faced in treating lung diseases. However, limited success has been recorded in the industrialization translation of inhalable nano-formulations. Only one relevant product has been approved by the FDA to date, suggesting that there are still many issues to be resolved in the clinical application of inhalable nano-formulations. These systems are characterized by a dependence on inhalation devices, while the adaptability of device formulation is still inconclusive, which is the most important issue impeding translational research. In this review, we categorized various inhalable nano-formulations, summarized the advantages of inhalable nano-formulations over conventional inhalation formulations, and listed the inhalable nano-formulations undergoing clinical studies. We focused on the influence of inhalation devices on nano-formulations and analyzed their adaptability. After extensive analysis of the drug delivery mechanisms, technical processes, and limitations of different inhalation devices, we concluded that vibrating mesh nebulizers might be most suitable for delivering inhalable nano-formulations, and related examples were introduced to validate our view. Finally, we presented the challenges and outlook for future development. We anticipate providing an informative reference for the field.

## 1. Introduction

Nano-formulations are diversely defined by different pharmaceutical scientists and drug regulatory agencies all over the world. The nano-formulations mentioned in this work refer to the nanoscale particles prepared from pure active pharmaceutical ingredients (APIs) using nano-formulation technology or nanoscale particles formed by combining APIs with appropriate carrier materials and the final pharmaceutical preparations thereof [1]. The final product or carrier material of nano-formulations has a particle size of typically less than 1000 nm, with significant scaling effects, and typically exhibits a well-defined physical interface [2].

The main types of nano-formulations are liposomes [3,4], polymer micelles [5,6], nanoemulsions [7,8], nanocrystals [9,10], etc. Compared with conventional formulations, nano-formulations may have the following potential: (i) increasing the solubility and bioavailability of the APIs or significantly reducing the food effect and inter-individual differences; (ii) increasing the stability of the APIs in vitro and in vivo; (iii) controlling the release profile of the APIs; (iv) improving the selectivity of the APIs to tissues, organs, or cells and thus enhancing the efficacy of the APIs and reducing adverse reactions; (v) offering new routes of drug delivery; and (vi) changing the physical status of the APIs. As a result, the convenience of clinical administration and the patient’s compliance can be elevated [2,11,12,13,14,15,16]. Nano-formulations are currently used in a variety of routes of administration, including intravenous [17], oral [18], transdermal [19], ocular [20], and pulmonary [21] delivery, for treating systemic and local diseases. In recent years, inhalable nano-formulations have generated a great deal of interest for the following reasons.

The global morbidity and mortality of respiratory diseases such as chronic obstructive pulmonary disease (COPD) and asthma have increased from 1990 to the present [22,23,24,25,26]. Despite the progress in drug discovery and clinical diagnosis, there is still a lack of effective treatments for these diseases. Over the past 50 years, the incidence and mortality rates of lung cancer have increased significantly, ranking as the first place of all malignant tumors in males and second place in females. More and more attention is being paid to the pathogenesis, diagnosis, and treatment of lung cancer [27,28,29,30,31]. In addition, recent outbreaks of respiratory infectious diseases such as COVID-19 have accumulated global research interests [32,33,34,35,36]. The difficulty in treating these respiratory diseases may be due to inadequate doses of drugs entering the respiratory tract or insufficient targetability to the lesion sites when conventional pharmaceutical preparations are used [37,38].

In this context, there has been a focus on new approaches to achieve more effective treatment of lung diseases, in which inhalable nano-formulations have attracted the interest of many researchers with the development of nanotechnology. Inhalable nano-formulations have the following advantages in respiratory disease therapy: (i) reducing the administration dosage [39,40]; (ii) increasing the solubility of the APIs [41,42]; (iii) achieving targeted drug delivery towards lung lesions [37,43]; (iv) API absorption across the epithelium can be enhanced [44,45]; and (v) enabling pulmonary retention [46,47]. Due to these advantages, nanotechnology can ensure the therapeutic efficacy of APIs in dissatisfactory situations where the patient’s condition (e.g., unconsciousness, insufficient inspiratory flow rate, breath-holding problems, and inadequate coordination with the use of inhalation devices) results in poor inhalation effectiveness. Therefore, inhalable nano-formulations are considered to have promising applications in treating COPD, asthma, lung cancer, COVID-19, and other lung diseases [23,42,48,49]. 

**Figure 1:**
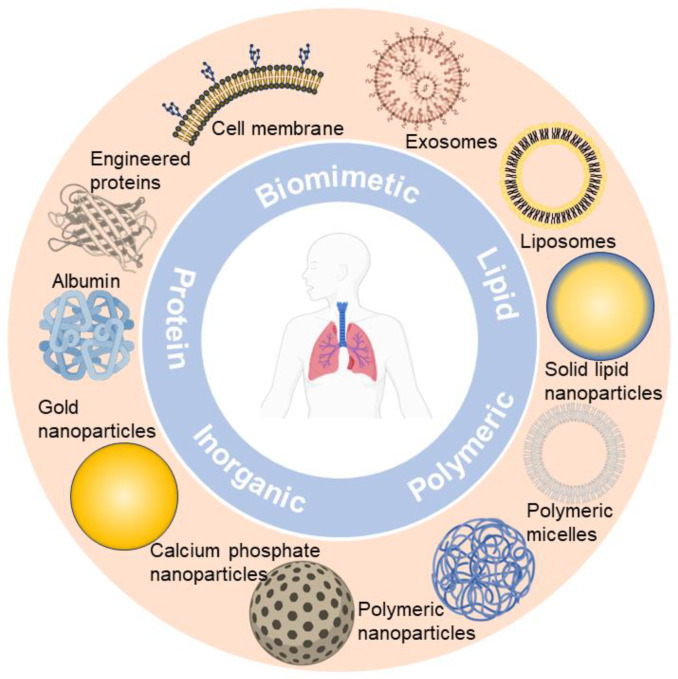
Representative nano-formulations in pulmonary drug delivery.

Up till now, nano-formulations investigated for pulmonary drug delivery (summarized in Figure 1) mainly include polymeric nanocarriers (e.g., polymeric nanoparticles and polymeric micelles) [50,51], lipid-based nanocarriers (e.g., liposomes and solid lipid nanoparticles) [4,52], protein-based nanocarriers (e.g., albumin and engineered proteins) [53,54], inorganic nanocarriers (e.g., gold nanoparticles and calcium phosphate nanoparticles) [55,56], and biomimetic nanocarriers (e.g., cell membrane and exosomes) [42,57,58]. We observe that nano-formulations are booming in the field of pulmonary delivery, and a diversity of nano-formulations can be regarded as drug candidates for respiratory disease treatment. Ultimately, nano-formulations are incorporated into inhalation devices (e.g., nebulizers, dry powder inhalers (DPIs), metered dose inhalers (MDIs)) for treating respiratory disease, either by themselves or with excipients to form solid particles [37].

This review will provide an overview of four aspects: fundamental and industrial translational research on inhalable nano-formulations, common inhalation devices, and inhalation devices suitable for nano-formulations. We focus on exploring the translational aspects of inhalable nano-formulations and making recommendations accordingly. This review is anticipated to provide some understanding for the subsequent industrial translation of inhalable nano-formulations.

## 2. Fundamental Research on Inhalable Nano-Formulations Is Comprehensively Conducted

**Figure 2:**
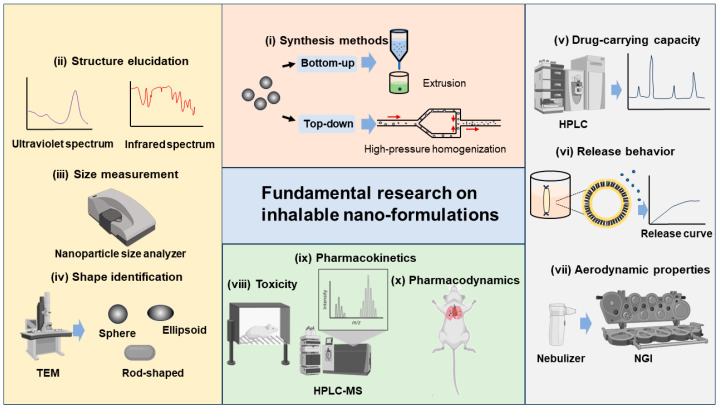
Fundamental research paradigm of inhalable nano-formulations.

Regarding the current study of inhalable nano-formulations, the physicochemical, pharmaceutical, and toxicological properties have been intensively studied, which can be categorized into fundamental research. We believe that the firm knowledge acquired from fundamental research is the key basis of industrial translation. The well-documented fundamental research paradigm is depicted in Figure 2 and summarized as follows:

(i) Synthesis methods. There are various synthesis methods available for different inhalable nano-formulations, which can be broadly categorized into top–down and bottom–up methods. Top–down methods refer to the decomposition of larger solid particles into smaller nanoparticles by external forces, such as high-pressure homogenization and wet milling. Bottom–up methods refer to the synthesis of nanoparticles from the molecular level by precipitation, crystallization, and the removal of solvents, such as extrusion, solvent evaporation, and antisolvent methods [4,59,60,61,62].
(ii) Structure elucidation. For synthesized nano-formulations, we need to determine the nanoarchitectonics, including the atomic, molecular, nanoscale, and mesoscale structures. Techniques such as ultraviolet spectrum, infrared spectrum, nuclear magnetic resonance spectroscopy, mass spectrometry, X-ray diffraction, and X-ray photoelectron spectroscopy can be utilized [63].
(iii) Size measurement. The size distribution of nano-formulations is commonly studied by nanoparticle size analyzers. The main tests include PDI, size distribution, and autocorrelation function (ACF) curves, to ensure that the size of the prepared nano-formulations meets the inhalation requirements. After nano-formulations have been prepared into forms suitable for use in inhalation devices such as DPIs, nebulizers, and others, it is equally necessary to study their particle size, PDI, and other properties, to ensure that the sprayed droplets or dry powders meet the size requirements for effective lung deposition [45,64,65].
(iv) Shape identification. The shape of nano-formulations is commonly studied using a Transmission Electron Microscope (TEM), a Scanning Electron Microscope (SEM), and an Atomic Force Microscope (AFM). In addition to the static shape, it is vital to study the shape of sprayed droplets or dry powders after administration to observe the corresponding changes. The shape of xenobiotic particles affects the physiological behavior. For instance, elongated or rod-shaped particles are difficult to be phagocytosed by macrophages, while spherical or elliptical particles have a stronger targeting effect on macrophages [66]. If we can better understand the interplay between the particle shape and the cells, it will help us to develop pulmonary drug delivery systems with greater targetability [67].
(v) Drug-carrying capacity. The large specific surface area or accommodation room of nanoparticles allows for the surface adsorption or physical encapsulation of drug molecules, leading to a better drug-carrying capacity compared to formulations with larger particles [68,69]. The drug-carrying capacity of nano-formulations is commonly analyzed by High-Performance Liquid Chromatography (HPLC) or HPLC tandem methods.
(vi) Release behavior. For the release profile of inhalable nano-formulations in the lungs, they are generally expected to endow a rapid and complete one after deposition in the lungs, thus improving bioavailability [70]. The release behavior of nano-formulations is often studied in vitro by simulating the in vivo environment (like stimulating lung fluid), and release curves are drawn for analysis.
(vii) Aerodynamic properties. It is generally believed that the optimal aerodynamic size range for inhalable particles is 1–3 μm, with which a satisfactory lung deposition can be achieved [64]. From this viewpoint, compared with nano-formulations, micron-sized formulations have superior aerodynamic properties, and thus, current studies seek to enlarge the aerodynamic diameter of the nano-formulations through microencapsulation or bulking techniques, without affecting the excellent bioavailability of the nano-formulations. The Next-Generation Impactor (NGI) is commonly used to study the aerodynamic properties of nano-formulations. Via the NGI, parameters such as fine particle dose (FPD), fine particle fraction (FPF), mass median aerodynamic particle diameter (MMAD), and geometric standard deviation (GSD) can be determined as indicators of aerodynamic performance [71,72].
(viii) Toxicity. The toxicity of nano-formulations mainly stems from two aspects, APIs and nanocarriers. In the area of pharmaceutics, nanocarrier toxicity is emphasized. Smaller nanocarriers are difficult to phagocytose by macrophages and are thus retained in the alveoli, which may produce side effects [73]. In addition, the residual organic solvents and metal ions remaining in the formulations may cause inflammatory and other adverse reactions [74,75]. We need to conduct in vitro and in vivo toxicity testing of the nano-formulations, to assess the safety [72]. Interestingly, it is pointed out that the precise targeting of the nano-formulations can prevent the non-specific interactions between nano-formulations and lung cells and thus alleviate local toxicity [76].
(ix) Pharmacokinetics. By improving the pulmonary deposition rate of nano-formulations as well as active targeting modifications, the pharmacokinetics properties may be enhanced [42]. High-Performance Liquid Chromatography–Mass Spectrometry (HPLC-MS) is commonly used for pharmacokinetic-related studies. Inhalable nano-formulations in the lungs should be concerned with the absorption, distribution, metabolism, and excretion processes [37,70].
(x) Pharmacodynamics. The efficacy of nano-formulations can be enhanced by designing and screening the optimal formulation and administration scheme [42]. Pharmacodynamic studies of nano-formulations in pulmonary delivery are conducted in vitro to investigate whether the formulations produce the desired effects on cells (e.g., killing cancer cells, regulating gene expression and production of anti-inflammatory factors, etc.). In vivo, experiments were conducted to investigate whether the formulations could treat animals with lung diseases [28,40,45,49,77].

## 3. Industrial Translation of Inhalable Nano-Formulations Is Limited

As mentioned above, in recent years, inhalable nano-formulations have been widely subjected to fundamental research, while translational research on inhalable nano-formulations is still in a latency period. Currently, there is only one marketed product and a few inhalable nano-formulations progressing into clinical trials (Table 1). In particular, the only marketed inhalable nano-formulation is the FDA-approved product ARIKAYCE^®^ (an amikacin liposome suspension) inhalation suspension in 2018. It must be borne in mind that, for the research and development of drug formulations, it only makes sense to plunge into industrialization with a commercially available product. From this standpoint, there is still a long journey for the translation of inhalable nano-formulations.

Confronting this situation, we will systematically analyze how nano-formulations for inhalation can be industrially and clinically translated.

Most importantly, we should recognize that inhalable nano-formulations belong to the pulmonary drug delivery system, and thus, the requirements of such a delivery system should be met. All inhalable formulations have a corresponding inhalation device for administration. In other words, inhalation drug delivery systems are characterized as “drug and device in one”, meaning that inhalation formulations and their inhalation devices are mutually dependent [87,88,89]. Similarly, inhalable nano-formulations require a suitable inhalation device, which is de facto the focus of the translation bottleneck.

However, there is a lack of definitive research on what delivery devices are appropriate for inhalable nano-formulations [37]. In brief, different inhalation devices have different effects on the aerosolization performance, and hence, when choosing an inhalation device for inhalable nano-formulations, we need to study the aerosolization performance accordingly, to find the most suitable inhalation device. When we examine the adaptability of an inhalation device for the designed inhalation formulation, parameters as evaluation indexes like FPD, FPF, MMAD, GSD, drug deposition, drug delivery rate, total amount of delivery, and delivery dose homogeneity should be taken into consideration. In other words, an inhalation device that is compatible with an inhaled formulation should ensure a suitable and uniform aerodynamic diameter, a good lung deposition rate, and rapid delivery to the site of action, as well as adequate and quantitative dosage [90,91,92,93,94]. 

## 4. Various Inhalation Devices Are Used Clinically

**Figure 3:**
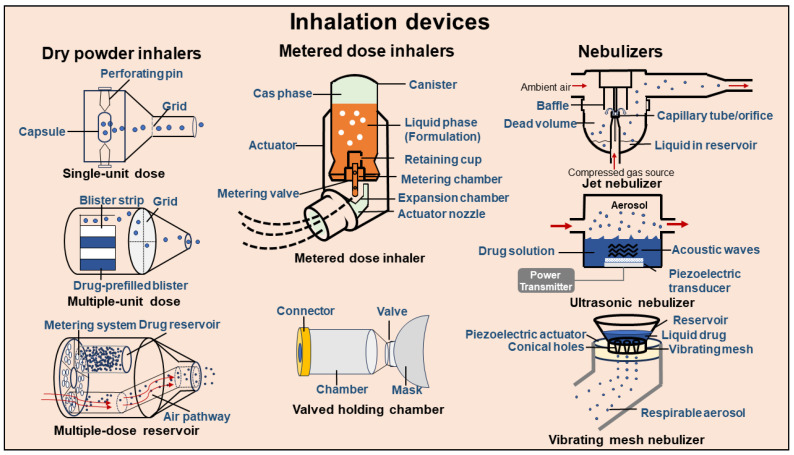
Common inhalation devices.

The most commonly used clinical inhalation devices can be divided into three categories (Figure 3): MDIs, DPIs, and nebulizers [37,42]. These devices have different delivery mechanisms and therefore are adaptable to different formulations. In addition, the physical condition of the patient has an impact on the choice of inhalation device. Unconscious patients should not use inhalation devices that require active inhalation, such as MDIs, but should use inhalation devices that can be administered through natural breathing, such as nebulizers [95]. In patients with severe lung diseases, the choice of inhalation device needs to consider the patient’s ability to achieve a sufficient inspiratory flow rate as well as the patient’s breath-holding problems to ensure an effective dose [45]. Similarly, the selection of an inhalation device needs to take into account the patient’s ability to coordinate adequate maneuvers to achieve a therapeutic effect [64]. Their definitions, characteristics, advantages, and limitations are described in detail below (Table 2).

**MDIs**: MDIs are a type of inhalation device that is widely used in clinical practice today in which drug-containing solutions, emulsions, or suspensions are encapsulated in pressure-resistant containers with special valves along with a suitable propellant. The energy provided by the ejection of propellant is used to form and release an aerosol [42,64]. Currently, the propellant used in MDIs has been replaced by the more environmentally friendly hydrofluoroalkanes (HFAs) from the previously used chlorofluorocarbons (CFCs) [96]. Due to the fast initial flow rate of the aerosol ejected from the nozzle in the MDI, the patient needs to have good coordination between inhalation and ejection maneuvers, but it is more difficult for children, elders, and disabled patients to correctly complete these maneuvers. In addition, the deposition rate of the sprayed drug from the MDI in the lungs is relatively low (only 10–20%) [37,95]. Therefore, to increase the effective deposition, an inhalation-aiding apparatus—a valved holding chamber (VHC)—between the MDI and the mouthpiece (or mask) is designed, which creates a buffer space between the MDI and the patient’s mouth and reduces the airflow. The VHC reduces the velocity of the aerosol and prolongs the vaporization time of the propellant, negating the need for inhalation–ejection coordination and increasing the rate of drug deposition in the lungs [97,98,99]. In addition, many researchers are also working on better-performing propellant agents that will improve the lung deposition rate of drugs and protect the environment [100,101].

**DPIs**: DPIs are powdered drug-containing particles of propellant-free formulations, which are driven into the airway by the airflow of the patient’s inhalation to overcome the trouble of actuation and inhalation coordination. DPIs are formulated in the pattern of solid micronized APIs or dry powder forms of APIs either alone or in combination with suitable carriers, which are subsequently loaded into a specific device in the manner of capsules, blisters, or multi-dose reservoirs. DPIs are categorized into single-dose, multi-unit dose, and multi-dose types. The rate of drug deposition in the lungs varies considerably between products, typically 12–40% [37,93,102,103]. DPI formulations are of solid state, stable for long-term storage, and easy to transport. In addition, DPIs are less likely to pollute the environment without propellants. The main drawbacks of DPIs are the high cost and that the delivery efficacy is dominated by the patient’s inhalation ability and speed [42,49]. In recent years, the improvement in DPIs is mainly reflected in simplifying the device structure, lowering the peak inspiratory flow rate requirements, increasing the dose uniformity, and upgrading the inhalation feedback function [95,104].

**Nebulizers**: Nebulizers are devices that use compressed gas (air or oxygen), ultrasound, and electric shock to provide energy to convert a drug-containing solution or suspension into an aerosol, which can be inhaled through a mask. They are divided into three main types: jet nebulizers, ultrasonic nebulizers, and vibrating mesh nebulizers [37,95]. When nebulization is performed, the patient can inhale the aerosol through calm breathing, and no special inhalation technique is required, which is suitable for geriatric, pediatric, and unconscious patients [42]. However, nebulized inhalation treatment consumes a longer time, resulting in vulnerability to contamination [105].

Most of the clinically used formulations for nebulization use a jet nebulizer, which uses compressed gas to nebulize the drug-containing solution or suspension to nebulized droplets with a diameter of less than 10 μm. It can further be divided into velocity-modifying type, breath-enhanced type, and breath-actuated type [86,97,98]. Jet nebulizers nebulize drugs based on the Venturi effect and Bernoulli’s principle and are suitable for delivering solutions and suspensions and can deliver antimicrobials, liposomes, and recombinant cells that cannot be delivered by MDIs and DPIs [106]. In addition, jet nebulizers are cheap and easy to use, making them the most commonly used type of nebulizer [37]. However, jet nebulizers take a longer time to nebulize, are not portable, and are noisy. They have a large amount of drug residue, and even if the solution or suspension is fully nebulized, some of the liquid adheres to the inner wall, thus requiring a larger dose and a low lung deposition rate of the nebulized drug (approximately 10%) [37,107]. Newer jet nebulizers (such as the breath-enhanced Pari LC^®^ Star, PARI, Starnberg, Germany) have improved these problems with mechanical adjustment technology that reduces aerosol loss and shortens nebulization time [90]. But, in general, jet nebulizers are technically crude and need further improvement. 

In ultrasonic nebulizers, through a high-frequency alternating electric field induced by the piezoelectric transducer, the electrical signal is converted into a periodic mechanical vibration. Through the coupling liquid, the periodic mechanical vibration is transferred to the drug-containing liquid, inducing the drug molecules to vibrate, and ultimately leading to the liquid interface rupture and the production of aerosol droplets. The rate of nebulization and the size of the droplets can be adjusted according to the patient’s condition [108,109]. Compared with jet nebulizers, ultrasonic nebulizers have a tighter structure and a faster drug delivery velocity, with a higher rate of drug deposition in the lungs [37,110,111]. However, the aerosol density produced by ultrasound is high, and the partial pressure of oxygen in the respiratory tract after inhalation is relatively low, making it unsuitable for patients with hypoxia or hypoxemia [109]. In addition, during ultrasonic nebulization, the liquid heats up, evocating solvent. Under this circumstance, the ultrasonic nebulizers may disrupt the chemical structure of biomolecules and thermolabile drugs [37,109] and cause the agglomeration or Ostwald ripening of particles in the suspension, leading to a higher incidence of adverse reactions [62,64]. Therefore, it is not suitable for suspensions and protein drugs.

Vibrating mesh nebulizers are a new generation of nebulizers that are similar to ultrasonic nebulizers in that they also use ultrasound to generate aerosols, but their operating principles are completely different from those of traditional ultrasonic nebulizers [49,112]. In the vibrating mesh nebulizer, there are thousands of conical-shaped stainless-steel meshes with micropores of about 3 μm in diameter, whose bottoms face the drug-containing liquid. The pore size can be adjusted according to the specific requirements. Upon ultrasonic vibration, the liquid is extruded, resulting in many droplets, which can be divided into active vibrating mesh nebulizers and passive vibrating mesh nebulizers [105,113]. Although vibrating mesh nebulizers are expensive and require regular maintenance and cleaning [114,115], we believe they are promising pulmonary delivery devices for clinical application.

In conclusion, different inhalation devices have different characteristics, advantages, and disadvantages, and which are more suitable for inhalable nano-formulations needs to be analyzed in terms of the mechanism of drug delivery.

## 5. Vibrating Mesh Nebulizers Are Suitable for Inhalable Nano-Formulation Delivery

A dominant reason for the deficiency in the commercially available nano-formulations for inhalation is the lack of a suitable inhalation device. As mentioned above, in pulmonary drug delivery, the optimal aerodynamic diameter of the aerosol should range from 1 to 3 μm, where the drug is efficiently deposited in the lungs [42,63]. There are currently two methods for the pulmonary delivery of nano-formulations to achieve this aim: (i) nebulization of nano-formulations by generating micron-sized droplets [116,117,118] and (ii) aerosolization of solid microscale powders containing nano-formulations (e.g., nano-agglomerates) [37,119,120]. Many studies have confirmed that the first approach is much simpler in terms of industrialization and has less impact on the physicochemical properties of the nano-formulations, while the second approach is more challenging [62,90,100,107,121]. In the following section, we will analyze the mechanism of drug delivery by inhalation devices and propose suitable inhalation devices for nano-formulations.

### 5.1. Status of Research on Using DPIs to Deliver Inhalable Nano-Formulations

The mechanism of drug delivery by DPIs is that the microscale powder consisting of the drug and carrier (generally lactose) is dispersed into the lungs in the form of an aerosol, through turbulence generated by the patient’s inhalation and the internal resistance of the device [93]. From this angle, we can interpret that the main issues to be examined in the DPIs for nano-formulations are (i) whether the nano-formulation is affected in the drying process; (ii) how the nano-formulation forms solid microscale powder and whether it can be effectively deposited in the lungs; and (iii) whether the solid microscale powder deposited in the lungs can be dispersed in the initial nano-formulation form. 

For issue (i), dry powders of inhalable nano-formulations need to be prepared by drying, and the main drying methods are categorized as freeze drying, spray drying, and spray–freeze drying [119]. Freeze drying is a commonly used drying method to improve the stability of perishable drugs (e.g., proteins, vaccines, etc.). Typical freeze drying is divided into three stages: freezing, primary drying, and secondary drying. Firstly, the system is frozen, thus separating most of the solvent from the nano-formulation. Secondly, the solvent is sublimated under low-pressure and low-temperature conditions. Finally, low-pressure conditions are maintained, and the temperature continues to be raised to remove all the solvents [122]. However, during the freezing process, the nano-formulation may be unstable, leading to irreversible aggregation, and the particle size of the dry powder is difficult to control [123], and therefore, freeze-drying technology is not suitable for inhaled nano-formulation. Spray drying is a drying method for thermal-stable formulations. It consists of three stages: atomization, dehydration, and powder collection. Firstly, the fed formulations and drying adjuvant are atomized into droplets. Secondly, the droplets are dried by heated air. Finally, dried nano-powders are collected [124]. Spray drying allows for the precise control of the particle size of the dry powder and a short processing time [119]. However, due to the process of atomized droplet drying, the parametric issues of spray drying (e.g., feeding rate and drying kinetic [125]) may affect the aerodynamic and dispersing properties of the dry powder [126]. These parameters may require the extensive design of experiments (DoE) to optimize [125]. In addition, spray drying is also not applicable to thermolabile drugs and has a relatively low yield [127]. Therefore, spray-drying technology is relatively costly and may only apply to a few inhalable nano-formulations, which are risky in industrialization. Spray–freeze drying is a relatively new technology in the pharmaceutical industry, combining freeze drying and spray drying by freezing atomized droplets plus lyophilizing [122]. Spray–freeze drying is suitable for thermolabile drugs due to the avoidance of thermal stress and has a similar way of controlling the particle size by modulating parameters to spray drying [124]. However, properties like the dispersibility of products collected from spray–freeze drying have been reported to be unsatisfactory, and the yield is lower compared to ordinary spray drying [119]. Therefore, spray–freeze drying may be less suitable for the industrialization of inhalable nano-formulations. To summarize, the current drying techniques are less adaptable for fabricating nano-formulation-based DPIs.

For issues (ii) and (iii), inhalable nano-formulation dry powders are mainly categorized into nano-embedded microparticles and nano-agglomerate microparticles [119]. Nano-embedded microparticles are nano-formulations encapsulated or dispersed into a micron-sized matrix. The microparticle is formed by encapsulating or dispersing the nano-formulation within a matrix of a bulking agent or shell-forming agent, during drying [128]. After the nano-embedded microparticles are deposited in the lungs, the matrix is degraded by the tissue fluid, releasing the nano-formulation [129]. However, the formation of the matrix requires a large number of excipients, resulting in a low API loading capacity, while the lung deposition rate varies widely, making the API dosage difficult to control [95,130]. Nano-agglomerated microparticles (also known as Trojan microparticles [131]) are formed by the drying of nanoparticles to reduce the huge surface energy. Electrostatic interactions and van der Waals forces take part in the polymerization process [119]. Protective agents are added during the drying process to ensure that the nano-formulation is not destroyed and can be effectively redispersed after the microparticles are delivered [132]. However, the commonly used protective agent, lactose, affects the flowability and aerosol properties of the powder after drying [119]. In addition, in terms of industrialization, it is difficult to ensure the batch-to-batch homogeneity and stability of the nano-aggregated particles after the drying process [133,134]. To conclude, the stability of microparticles and the reconstitution of nanoparticles cannot be guaranteed in nano-formulation-based DPIs.

Overall, the current practice of DPI design and development does not provide a good solution to the various above-mentioned problems that would be encountered in the industrialization of inhalable nano-formulations, and the solution to these problems is still challenging. In particular, the drying aspect imposes great difficulties for industrialization, with high costs and risks. In addition, the dry powder inhaler itself needs to be further optimized concerning the wide variation in lung deposition rates [135]. Therefore, for the time being, DPIs are not very suitable as inhalation devices for the industrialization of inhalable nano-formulations, but it is believed that with the development of new technologies (e.g., nano-spray-drying technology [127] and supercritical fluid drying technology [136]), DPIs may be able to be used in the future as inhalation devices for nano-formulations.

### 5.2. Status of Research on Using MDIs to Deliver Inhalable Nano-Formulations

The drug delivery mechanism by MDIs is that the ejection of propellant co-encapsulated with the drug-containing system provides energy to form and release an aerosol for delivery [64]. We have considered the following issues and concluded that MDIs may not be well suited for the pulmonary delivery of inhalable nano-formulations. Firstly, the coordination of the actuation and inhalation of MDIs is somewhat difficult, resulting in a large variation in the lung deposition rate, making it tough to control the dose of nano-formulations [37]. Secondly, the main propellants applied in MDIs are HFAs, which are organic solvents with a certain solubility for a spectrum of nano-formulations, having the potential to disrupt the nanostructures, thus affecting the stability [90]. In addition, the high shear stress when MDIs release aerosols may likewise disrupt the nanostructure. The destruction of nanostructures can lead to the leakage of loaded APIs. Thus, the stability of the APIs may also be affected, leading to a decrease in bioavailability, and ultimately, the APIs may not be able to achieve the desired therapeutic effect [64]. Thirdly, ethanol, which is commonly used as a co-solvent to improve the stability of nanosuspensions in MDIs, has also been reported to affect the performance of aerosols [90]. In addition, the choice of propellants and co-solvents affects the evaporation kinetics, thus affecting the delivery efficacy of MDIs [42]. Fourthly, for large-dose nano-formulations, the flocculation phenomenon may occur, which affects the accuracy of drug delivery [90]. 

In summary, the use of MDIs for the pulmonary delivery of inhalable nano-formulations may need more investigation, especially in terms of propellant-relevant issues. How to leave the least impact on the stability of the nano-formulation and improve the drug deposition rate in the lungs is something that needs to be explored in depth [137].

### 5.3. Status of Research on Using Nebulizers to Deliver Inhalable Nano-Formulations

The mechanism of drug delivery by nebulizers is that an aqueous solution or suspension of an API is nebulized into an aerosol, which is delivered to the lungs by nebulization [64]. Compared with MDIs and DPIs, nebulizers can directly turn the configured system into an aerosol without excessive processing, which has less impact on the original nano-formulation and is more advantageous in drug development [37]. Consequently, we initially believe that nebulizers may be more suitable as inhalation devices for inhalable nano-formulations.

Firstly, for formulation development, nebulizers do not require complex formulations and can directly nebulize configured solutions and suspensions, or even intravenous formulations, which greatly reduces the difficulty of formulation screening [42]. For nebulized nano-formulations, the excipients used are very simple. Besides nanocarrier excipients, only suspending aids are used, which have essentially no effect on the nebulization process of the nano-formulation. Compared to DPIs and MDIs, the formulation is much simpler [138]. In addition, these systems reduce or minimize irritation and the potential toxicity of the formulation due to the low organic solvent content [62]. They are generally prepared as nanosuspensions, using the top–down approach or the bottom–up approach [62]. In particular, nebulizers are effective in simplifying the preparation procedures compared to MDIs and DPIs. A variety of methods can be chosen for the preparation of nanosuspensions for nebulization [62]. Also, nanosuspensions are easier to nebulize and have better lung deposition rates compared to conventional formulations because the aggregates of the nano-formulations in the droplets have superior aerodynamic diameters [139]. The nebulization of nanosuspensions into micron-sized droplets is undoubtedly the easiest way to deliver nano-formulations into the lungs [37]. Chiang et al. compared the nebulization performance of plain and nanosuspensions of fluticasone. They found that the nanosuspensions were not only very stable in terms of nebulized drug delivery but also had good pulmonary deposition rates and lower systemic exposure. They concluded that nebulized nanosuspensions were well suited for pulmonary drug delivery [139]. Similar observations were made by Wiedmann et al., who found that nanosuspensions of beclomethasone dipropionate had better pulmonary deposition rates after nebulization compared to micronized suspensions [140].

Secondly, the adaptability is determined by the therapeutic efficacy of the developed device–formulation combination. And it should be safe enough not to produce strong irritation or serious toxic side effects on the body. Patlolla et al. encapsulated celecoxib (Cxb) in nanostructured lipid carriers (Cxb-NLCs) and evaluated the deposition of nebulized Cxb-NLCs in the lungs of mice. Their results showed good FPF and MMAD of Cxb-NLCs. The FPF of the drug was four times higher than that of the nebulized celecoxib solution and had better aerodynamic properties, higher bioavailability, and good stability [141]. Meng et al. designed a dexamethasone neutrophil nanoparticle (nanoDEX) for the treatment of lung inflammation and injury by nebulized delivery, thus providing therapy for COVID-19 and other respiratory diseases with new strategies. Their study showed that the treatment of lung inflammation and injury in non-human primates with nanoDEX by nebulization was equivalent to the efficacy of a 10-fold dose of intravenously administered dexamethasone and that the lung deposition rate of nanoDEX was 14-fold higher as compared to dexamethasone nebulization [142]. Lokugamage et al. designed and optimized lipid nanoparticles (LNPs) to efficiently deliver mRNA to the lungs by nebulization, thereby preventing influenza A (H1N1). Their study showed that the mRNA delivered by the nebulization of the screened LNPs was more effective in preventing H1N1 in mice than systemic administration [143]. Walter et al. used suspended nanocrystals of budesonide for the nebulization treatment of cholesterol-responsive lung diseases (e.g., asthma). Their results showed faster drug delivery, shorter duration of nebulization, and fewer adverse effects compared to an FDA-approved nebulization treatment with inhaled cortisone suspension (Pulmicort Respules^®^, AstraZeneca, London, UK). In addition, the proposed system greatly improved the bioavailability and intrapulmonary distribution of budesonide [144]. Overall, nebulizers are the inhalation devices often chosen by researchers both in preclinical and clinical studies of inhalable nano-formulations. The nebulizer can not only have little impact on the physicochemical stability, facilitate formulation screening, and exert a good therapeutic effect but can also exhibit a high safety profile and fewer adverse reactions. Therefore, nebulizers are very suitable as the inhalation device for inhalable nano-formulations in terms of industrialization.

Finally, as there are many types of nebulizers available, type selection is an important issue. As established in the previous section, because ultrasonic nebulizers do not apply to suspensions, they are not suitable as inhalation devices for nano-formulations, whose aqueous systems are usually in suspension form. Jet nebulizers, although mechanistically compatible with the delivery characteristics of nano-formulations, require further improvement regarding the effect of nebulization shear stress on nano-formulations, aerosol output, and patient compliance [105,145]. Therefore, current jet nebulizers are also less suitable as inhalation devices for nano-formulations.

As a new generation of nebulizers, vibrating mesh nebulizers solve the major problems faced by traditional nebulizers. They have many advantages, such as efficient drug delivery, short nebulization treatment time, very low API residue, low power consumption, quiet use, ease of carry, and so on. They are considered to greatly improve patient compliance and the effectiveness of the formulation [105,106]. Many researchers have experimentally demonstrated the advantages of vibrating mesh nebulizers as inhalation devices for inhalable nano-formulations compared to other nebulizers. Beck-Broichsitter et al. compared the aerodynamic characteristics of nebulizing nanoparticles by jet, ultrasound, and vibrating mesh nebulizers. Their results showed that vibrating screen nebulizers are more promising for nanoparticle inhalation applications [146]. Taylor et al. evaluated the aerosol properties and the stability of liposomes nebulized by a jet nebulizer (Pari LC^®^ Star) and a vibrating mesh nebulizer (Aeroneb Pro-8 μm). Their study showed that the vibrating mesh nebulizer was less destructive to liposomes and had a higher output efficiency [147]. Beck-Broichsitte et al. similarly concluded that nebulizing nanosuspensions was an appropriate method for delivering nanoparticles to the lungs and that vibrating mesh nebulizers were more efficient at delivering intact nanoparticles [116,148,149,150]. In addition, vibrating mesh nebulizers have given rise to a variety of nebulization technology subtypes, such as Aeroneb^®^ Pro and eFlow^®^, all of which have performed well in the pulmonary delivery of nano-formulations [151,152]. It is worth noting that the Lamira^®^ nebulizer system (using eFlow^®^), which is used in the FDA-approved and marketed ARIKAYCE^®^, was obtained by modifying vibrating mesh nebulizers [121]. Overall, vibrating mesh nebulizers should be the most suitable inhalation device available for inhalable nano-formulations.

**Figure 4:**
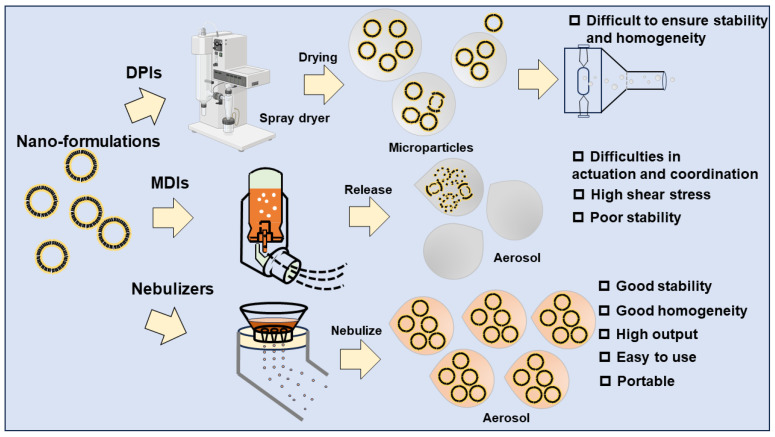
Adaptability of inhalation devices with nano-formulations.

In summary, by analyzing the mechanisms of different inhalation devices, we concluded that although all inhalation devices have their advantages/characteristics, for the up-to-date knowledge on inhalable nano-formulations, there is only one product on the market, ARIKAYCE^®^, which employs nebulizers as an inhalation device. Therefore, this review is intended to focus on the application of nebulizers, especially vibrating mesh nebulizers (Figure 4). Other inhalation devices are still promising as inhalation devices for inhalable nano-formulations in the future, with proper technological improvements. Next, we will preliminarily validate the statement by performing a brief analysis of ARIKAYCE^®^ which employs vibrating mesh nebulization technology.

### 5.4. Case Study of ARIKAYCE^®^

ARIKAYCE^®^ is the first and currently only formation approved by the FDA in 2018, specifically to treat Mycobacterium avium complex (MAC) lung disease. ARIKAYCE^®^ is chemically an amikacin liposome suspension that is nebulized for oral inhalation, in conjunction with a Lamira^®^ nebulizer (PARI, Starnberg, Germany). It is also the only marketed nano-formulation for the treatment of lung disease [121]. The recommended dosage of ARIKAYCE^®^ in adults is once daily or one 590 mg amikacin/8.4 mL ARIKAYCE^®^ vial. It is simple to administer, as follows: mixing the vial contents thoroughly, pouring the contents into the reservoir of the nebulizer handle, and finally starting the nebulizer for inhalation [153,154].

The formulation for ARIKAYCE^®^ consists of amikacin sulfate encapsulated in liposomes at a targeted concentration of 70 mg amikacin/mL, with a pH range of 6.1–7.1 and lipid–amikacin weight ratio in the range of 0.60–0.79. Inactive components include cholesterol and dipalmitoylphosphatidylcholine (DPPC), the lipid materials of liposomes, as well as sodium chloride to regulate osmolality, sodium hydroxide to regulate pH, and water for injection as a solvent [153,154]. Overall, the formulation for ARIKAYCE^®^ is relatively simple and only consists of common pharmaceutical excipients, which is consistent with the idea that excipients for nebulized formulations are simpler and safer, as we mentioned above.

The Lamira^®^ nebulization system used in ARIKAYCE^®^ is a nebulizer using eFlow^®^ technology, which is an active vibrating mesh nebulizer. The nebulization mechanism of this nebulizer is based on an electronic drive that causes the nozzle membrane to vibrate at a high frequency to generate acoustic pressure, producing individual droplets of regular size at the nozzle outlet. This type of nebulizer produces an inhalable aerosol with a narrow particle size distribution, virtually eliminating the API loss due to liquid adherence to the container surface in jet or ultrasonic nebulizers. In addition, the liquid is not subjected to repeated high shear stresses or heat during nebulization [155]. Standard in vitro testing of ARIKAYCE^®^ indicated that the average delivery dose is approximately 312 mg of amikacin sulfate (53%). The MMAD of the nebulized aerosol droplets, measured using the NGI method, was approximately 4.7 μm [153,154]. 

Moreover, clinical studies demonstrated that ARIKAYCE^®^ in combination with guideline-based therapy (GBT) significantly increased the probability of achieving sputum culture conversion (defined as 3 consecutive months of negative MAC sputum cultures) at month 6 in adult patients with refractory MAC lung disease, compared to GBT alone. ARIKAYCE^®^ treatment in combination with GBT resulted in a significantly higher retention rate of conversion responses than GBT-only treatment up to 12 months post-treatment [21,79].

Overall, ARIKAYCE^®^ can effectively boost the therapeutic effects of amikacin liposomes by nebulization to treat patients with MAC lung disease. ARIKAYCE^®^-related studies also demonstrated that vibrating mesh nebulizers have numerous advantages in serving as inhalation devices for inhalable nano-formulations and elucidated the feasibility of the translation of such nano-formulations.

## 6. The Development of Inhalable Nano-Formulations and Their Nebulizers Is Challenging

As technology advances, more attention is being paid to inhalable nano-formulations, and nebulization technology continues to evolve to match the various new formulations. Although inhalable nano-formulations have many advantages over traditional formulations, and nebulizers are suitable as inhalation devices of nano-formulations, we must realize that only one inhaled nano-formulation has been marketed so far, which indicates that there are still many major problems in the development of inhalable nano-formulations and their nebulizers.

Firstly, regarding the safety of nanomaterials, we must consider improving the safety of the nanomaterials used in industrialization. Liposomes, as the first FDA-approved nanocarriers, are good in biocompatibility and safety. However, for cationic liposomes to deliver sensitive compounds such as nucleic acids, there is still the problem of cytotoxicity, which needs to be further addressed to improve their safety [156]. Dendritic polymers increase the number of cations by branching with a number of terminal amino groups. Excessive cations can lead to cell membrane rupture and apoptosis, producing severe cytotoxicity [156]. The non-degradability of inorganic nanocarriers leads to their continuous accumulation in the reticuloendothelial system, which triggers inflammation and other adverse reactions [42]. Overall, during the design of inhalable nano-formulations, we recommend the use of FDA-approved nanomaterials, which will ensure the safety of the formulation and increase the feasibility of translation. In addition to the safety of nanomaterials, we should pay attention to the in vivo retention and distribution processes of nano-formulations when conducting fundamental research. For example, the retention of nano-formulations in the lungs, the absorption rate in the pulmonary capillaries, and the systemic side effects due to increased systemic exposure should all be taken into consideration [62]. All of these issues must be examined in the industrialization of inhalable nano-formulations.

Secondly, in the nebulization of nano-formulations, changes in nebulization parameters and related nano-formulation parameters can affect the nebulization process [37]. Therefore, to ensure an effective deposition rate, reduce adverse effects, and improve bioavailability, we need to consider both the nebulizer parameters and the physicochemical properties of nano-formulations. Active vibrating mesh nebulizers have low shear stress and low API loss [155]. They are equipped with electronic controls such as eFlow^®^ and AKITA^®^ APIXNEB systems, which optimize the nebulization process by controlling the flow rate, output, and time of the aerosol [90,155]. Parameters such as the osmotic pressure, pH, and viscosity of the nano-formulations can also affect the nebulization process and effective deposition rate. Low osmotic pressure may cause adverse reactions such as coughing and bronchoconstriction. High osmotic pressure stimulates respiratory secretion and may aggravate respiratory symptoms. In addition, the improper application of excipients that regulate osmotic pressure may affect the therapeutic efficacy and safety [37,157,158]. A high or low pH may lead to the leakage of the drug and adverse reactions such as bronchospasm and coughing in patients [157,159]. Increased viscosity may increase the nebulized droplet size, prolong the nebulization time, and disrupt the drug structure, thus reducing the output efficacy and affecting nebulizer operation [160,161,162]. Overall, the design of inhalable nano-formulations involves the choice of a reasonable inhalation device and the adjustment of the relevant parameters with suitable excipients to optimize the nebulization process and improve the effective deposition rate.

Finally, in large-scale production, we need to simplify the design of nano-formulations, consider issues such as cost, industrial equipment, etc., and pay attention to the introduction of relevant standards [2,37,163]. ARIKAYCE^®^, as mentioned above, is simple in design and production. The Lamira^®^ nebulization system used was modified for large-scale production. In addition, the online quality control equipment and some of the welding techniques were customized [121,155]. The industrialization of inhalable nano-formulations is also dependent on the formulation of standards by governmental departments and the unification of global standards. This will guide and promote researchers to carry out industrialized research on inhalable nano-formulations and achieve the purpose of large-scale production.

In conclusion, the development of inhalable nano-formulations and their nebulizers requires a detailed and feasible plan designed ab initio, and this plan needs to be continuously improved to meet the requirements for translation. Finally, the challenges may be gradually resolved to achieve large-scale production, through the collaboration of various fields.

## 7. Conclusions and Outlook

Inhalable nano-formulations are very promising as a means of treatment for lung diseases. Compared with traditional formulations, they have many advantages such as increasing solubility, improving bioavailability, and reducing toxicity. However, translational research on inhalable nano-formulations is scarce, and many unresolved issues render industrialization difficult. In this review, we summarized the advantages of nano-formulations for inhalation delivery and the current fundamental research paradigm, from which we found that the fundamental research on nano-formulations was relatively mature, but the translational research lagged far behind. We supposed that the main problem of such a situation was that the pulmonary drug delivery system needed cooperation between the inhalation formulations and the inhalation devices to deliver the drugs effectively. Therefore, we focused on analyzing the influence of inhalation devices on inhalable nano-formulations and what inhalation devices were suitable for inhalable nano-formulations. We believe that vibrating mesh nebulizers, a new-generation nebulizer, are more suitable for inhalable nano-formulations and offered some suggestions for the subsequent optimization. In addition, for the development of inhalable nano-formulations and their nebulizers, we summarized some of the problems faced. To achieve the goal of large-scale production, it was necessary to ensure not only the safety of nanomaterials and the optimization of formulations but also the maturity of industrial equipment and the introduction of relevant standards.

To date, only one inhaled nano-formulation, ARIKAYCE^®^, has been approved for marketing by the FDA, and there are a few clinical studies of such formulations. In addition, these studies are focused more on liposomes and less on other nano-formulations. All of this suggests that there are still issues with the translation of inhalable nano-formulations that need to be addressed, in terms of both inhalation devices and formulations. With the advancement in artificial intelligence (AI) technology, we can now monitor quality issues online, together with software analysis, which makes the optimization of the nebulization process easier [90]. We believe that more suitable nebulizers for nano-formulations as novel inhalation devices will appear soon, further promoting the translational research of inhalable nano-formulations. With the advancement in nanotechnology, more researchers are developing novel inhalable nano-formulations aimed at reducing adverse effects, increasing lung deposition rates, and addressing drug resistance [42]. In addition, improving the targeting ability of nano-formulations and overcoming physiological barrier hindrances are also the focus of research on inhalable nano-formulations. In particular, some researchers have proposed the use of microrobots to improve the targeting ability and overcome physiological barriers [164]. We believe that the continuous advancement in these new technologies will further accelerate the development of inhalable nano-formulations and lay the foundation for the industrialization of such formulations.

We hope that this review can provide some valuable suggestions for researchers to conduct translational research on inhalable nano-formulations and provide some help for the development of this industry. Lastly, the research and development of inhalable nano-formulations are rising, and we will conduct research in this area in the future. We believe that more inhalable nano-formulations will be marketed in the future to benefit patients with lung diseases.

## Figures and Tables

**Table 1:** Inhalable nano-formulations under clinical application or development for respiratory disease management.

Nano-Formulations	API	Implications	Status	Reference/NCT
Liposome	Amikacin	*Mycobacterium avium* complex (MAC) lung disease	Approved by FDA in 2018	ARIKAYCE^®^ KIT [21]
Liposome	Amikacin	CF	Phase III (last update posted in 2020)	NCT01316276 [78]
Liposome	Amikacin	NTM lung infection due to MAC	Phase III (last update posted in 2020)	NCT02344004 [79]
Liposome	Amikacin	Bronchiectasis	Phase II (last update posted in 2019)	NCT00775138
Liposome	Ciprofloxacin	Cystic fibrosis (CF)	Phase II (last update posted in 2014)	NCT00645788 [80,81]
Nano-vesicles/niosomes	Salbutamol Sulphate	Pulmonary disease	Phase I (last update posted in 2017)	NCT03059017 [82,83]
Gene product/lipid vector	pGM169/GL67A (Plasmid DNA)	CF	Phase II (last update posted in 2015)	NCT01621867 [84]
mRNA-refdLNP (not stated in the literature)	MRT5005 (CFTR mRNA)	CF	Phase I/II (last update posted in 2020, recruiting)	NCT03375047 [85]
Inhaled nanoparticle (not stated in the literature)	Remdesivir (GS-5734) and NA-831 (NEUROSIVIR)	COVID-19, SARS, Severe Acute Respiratory Syndrome, etc.	Phase I (last update posted in 2020, recruiting)	NCT04480333
Lipocalin-1	IL-4Ra antagonist (PRS-060)	Asthma	Phase I (last update posted in 2020)	NCT03574805 [86]

Data source: https://clinicaltrials.gov/ (accessed on 28 October 2023).

**Table 2:** Delivery mechanisms, advantages, and limitations of common inhalation devices.

Inhalation Devices	Delivery Mechanisms	Advantages	Limitations
DPIs	The drug-containing dry powder enters the airway through the airflow of the patient’s inhalation	✓ Propellant-free; ✓ Portable; ✓ No need to coordinate between inhalation and actuation; ✓ High inhalation efficiency; ✓ Disposable or reusable device; ✓ Wide range of devices.	◆ Passive inhalation; ◆ Pulmonary deposition of drugs is related to the patient’s ability and rate of inspiration; ◆ Expensive.
MDIs	Propellant provides energy to release drug-containing aerosols	✓ Portable; ✓ Easy to use; ✓ Wide range of applications; ✓ Cheap.	◆ Contains propellants that pollute the environment; ◆ Requires coordination between actuation and inhalation; ◆ Not suitable for a large dose; ◆ Low output; ◆ Low drug deposition in the lungs.
Jet nebulizers	Compressed air nebulizes drug-containing liquids into aerosols	✓ No need to coordinate between inhalation and actuation; ✓ Suitable for geriatric, pediatric, and unconscious patients; ✓ Delivery of large doses.	◆ Not portable; ◆ Noisy; ◆ High drug residues; ◆ Not easy to use; ◆ Low aerosol output; ◆ Not suitable for long-term treatment of chronic diseases.
Ultrasonic nebulizers	Nebulize drug-containing liquids into aerosols by generating high-frequency vibrations through a piezoelectric transducer	✓ No need to coordinate between inhalation and actuation; ✓ Suitable for geriatric, pediatric, and unconscious patients; ✓ Delivery of large doses; ✓ Require short treatment time; ✓ Higher rates of drug deposition in the lungs.	◆ Not portable; ◆ More expensive than jet nebulizers; ◆ Not suitable for delivery of highly viscous drugs or drugs that crystallize on drying; ◆ Not suitable for drug suspensions; ◆ Not suitable for patients with hypoxia or hypoxemia; ◆ Not suitable proteins and thermolabile drugs.
Vibrating mesh nebulizers	Drug-containing liquids are nebulized into aerosols by passing through ultrasonically vibrating mesh	✓ Portable; ✓ Silent; ✓ Easy to use; ✓ Require short treatment time; ✓ Suitable for unstable drugs; ✓ Low drug residues; ✓ High aerosol output; ✓ High rate of drug deposition in the lungs.	◆ Requires regular maintenance; ◆ Hard to clean; ◆ Not suitable for delivery of highly viscous drugs or drugs that crystallize on drying; ◆ Expensive.

## Data Availability

Details are available from authors.
